# From training to practice: A multi-group analysis of factors influencing K-12 teachers’integration of Digital Educational Resources (DERs)

**DOI:** 10.1371/journal.pone.0338543

**Published:** 2025-12-16

**Authors:** Qizhong Ou, Songqiao Wu, Yilu Jiang, Zhenni He, Haiqiu Luo

**Affiliations:** School of Artificial Intelligence, Nanning Normal University, Nanning, China; Shenzhen University, CHINA

## Abstract

Ensuring teachers effectively integrate digital technologies is crucial for modernizing education, yet the success of large-scale training initiatives often varies. This study investigates the factors shaping technology adoption among 2,821 K–12 teachers following a national digital literacy program. Using a Structural Equation Model (SEM) based on the UTAUT framework, this research employs a multi-group analysis to examine how adoption drivers differ across key demographic segments. Results show that social influence and performance expectancy are the primary drivers of teachers’ intention to use digital resources, which in turn strongly predicts actual use. Notably, the influence of these factors varies significantly depending on teachers’ regional context, educational stage, and prior digital experience, highlighting the limitations of a uniform approach to professional development. The findings provide a nuanced evidence base for designing more targeted and effective teacher support strategies.

## Introduction

The global education landscape is undergoing a rapid digital transformation [1]. Schools worldwide are integrating new technologies into their classrooms, making Digital Educational Resources (DERs) essential components of modern teaching and learning [2]. DERs are generally defined as educational materials in digital formats that teachers select to achieve various goals, such as transmitting content, enhancing learning experiences, developing skills, and assessing students [3,4]. These resources operate within a broader ecosystem supported by Information and Communications Technology (ICT), which provides the critical base for accessing, managing, and interacting with digital information [5]. Simply put, using ICT in an educational context means searching for, creating, and selecting DERs, as ICT is the pathway to access these resources [6]. The synergy between DERs and ICT enables teachers to move beyond simple knowledge transmission, fostering active, collaborative learning environments where students can develop crucial skills for the digital age [7,8].

Recognizing this potential, governments globally have launched strategic initiatives to promote the use of DERs. In China, for instance, initial policy support was established in 2017 with *The Guiding Opinions of the Ministry of Education on the Construction and Application of the Public Service System for Digital Educational Resources*, which aimed to advance education digitalization [9].More recently, China has updated and reinforced its commitment by issuing the *Opinions of the Ministry of Education and Nine Other Departments on Accelerating the Digitalization of Education* in 2025, which further emphasizes the vigorous construction and sharing of digital resources to accelerate the nation’s digital transformation [10]. Similarly, *The United States’ 2024 National Educational Technology Plan* focuses on closing digital access, design, and use gaps by ensuring equitable access to DERs, empowering educators to design technology-driven learning experiences, and enhancing students’ ability to effectively utilize digital tools [11]. *The European Union’s Digital Education Action Plan (2021–2027)* strategically promotes the development and integration of DERs to build a high-performance digital education ecosystem and enhance digital skills [12]. These parallel efforts underscore a global consensus on the pivotal role of DERs in developing high-quality education.

However, the success of these large-scale investments is not guaranteed. The ultimate effectiveness of DERs depends on their meaningful adoption and integration by K-12 teachers into daily classroom practice. Despite the widespread availability of these resources, a critical implementation gap persists. While these efforts reflect a global consensus, the implementation context in China presents a unique challenge and research opportunity. Compared to similar policies in other countries, China’s vast population base can easily lead to digital educational divides. Consequently, national education policies often place a greater emphasis on achieving equitable distribution of digital resources between regions (such as urban-rural gaps) and school types to promote educational equity [10]. This top-down, policy-driven environment provides a distinct setting to study technology adoption, where governmental initiatives create a powerful impetus for change. Yet, even with strong policy support, if the factors that affect teachers’ effective integration of digital educational resources are not clear, a large amount of public investment may still yield low returns [13]. As the OECD’s ‘2023 Digital Education Outlook’ report states, enabling wider audiences to benefit from digital education and ensuring equitable use of DERs will remain a major future challenge for governments [14].

To address this challenge, researchers have often turned to technology acceptance theories, with the Unified Theory of Acceptance and Use of Technology (UTAUT) being a prominent framework [15]. While existing meta-analyses of UTAUT [16] and Technology Acceptance Model(TAM) [17] offer valuable retrospective insights into the general drivers of educator’ technology adoption,they did not pay attention to the actual integration after specific large-scale vertical training interventions. More importantly, much of the existing research applying UTAUT [18] or UTAUT2 [19] in education tends to treat teachers as a single, homogenous group. This approach overlooks the profound heterogeneity within the teaching profession, yielding generalized findings that are insufficient for crafting targeted and effective policies. This oversight represents a significant gap in the literature: a lack of granular, evidence-based understanding of how technology adoption processes differ across distinct teacher subpopulations, especially after a uniform professional development experience.

This study aims to fill this critical gap by investigating the factors that shape K-12 teachers’ intentions and use of DERs immediately following a national, standardized digital literacy program. We applied the UTAUT framework and conducted group analysis by further expanding the moderating variables to unravel the internal complexity of the teacher group. Our research addresses the following questions:this study seeks to answer the following research questions:

**RQ1:** To what extent do performance expectancy, social influence, and facilitating conditions predict K-12 teachers’ behavioral intention and usage behavior of DERs after systematic training?

**RQ2:** How do the relationships in the UTAUT model differ across teachers with varying regional contexts, educational stages, and levels of digital technology experience?”

The pivotal contribution of this research lies in its large-scale, multi-group analysis that unpacks the complex heterogeneity of technology adoption in a post-training context. By analyzing data from 2,821 teachers, our study offers three distinct contributions. First, methodologically, it is different from traditional cross-sectional studies, we examine the crucial training-to-practice transition, a phase that is critical for policy success. Second, theoretically, we challenge the assumption of a uniform adoption process by demonstrating how key drivers are significantly moderated by regional differences, educational stage, and prior digital experience. Third, practically, the findings provide the precise evidence necessary to move beyond Traditional and universal insights toward targeted, context-sensitive interventions. This offers actionable insights for policymakers and educational leaders to design support systems that bridge the gap between policy goals and the actual integration by teachers in the classroom, thereby converting investments in DERs into tangible educational impact.

## Literature review

### Digital educational resources

To understand teachers’ technology adoption, it is essential to first clarify the technology in question. In the current educational landscape, DERs is a broad term for a wide array of digital tools and content. At its core, a DER is any learning or teaching material that exists in a digital format, designed to support instruction in both online and traditional classroom settings.

Within the specific context of this study, China’s national education policies provide a formal definition. The Standards for Content Review of Digital Educational Resources on the National Smart Education Platform define digital educational resources as fundamental components of the education system. These resources, designed specifically to serve educational purposes, are digitally processed collections used in teaching and learning. Examples include online courses, digital textbooks, e-books, teaching materials, case studies, virtual experiments and training, online teaching videos, and educational applications and tools [20]. The Teacher Digital Literacy education industry standard defines “digital technology resources” as digital education products used in teaching and learning. These include general software, subject-specific software, digital educational resources, smart education platforms, intelligent analysis and assessment tools, and smart classrooms [21].

The term DERs often serves as an umbrella concept for several related categories in international research [22]. For instance, Open Educational Resources (OERs), as defined by UNESCO, refer to freely accessible, openly licensed teaching and learning materials that can be used, adapted, and redistributed [23]. These include open textbooks, MOOCs, and freely available multimedia content, contributing to equitable access to education worldwide. Digital Learning Resources (DLRs) encompass a broad range of interactive, multimedia, and text-based digital content, such as e-books, simulations, virtual labs, and digital assessments, designed to support both online and traditional classroom instruction [24]. Furthermore, Learning Management Systems (LMS) such as Moodle, Blackboard, and Canvas serve as centralized platforms for organizing, delivering, and managing digital educational content [25]. These systems facilitate online learning, course management, and assessment administration. E-learning resources refer specifically to content developed for online education, including virtual learning environments (VLEs), recorded lectures, discussion forums, and gamified learning modules [26] Beyond content-focused resources, digital education technologies such as AI-driven adaptive learning platforms, intelligent tutoring systems, and data-driven analytics tools have gained prominence [2].

Given the diverse terminology in global and local contexts, this study adopts an inclusive definition of DERs. We define DERs as a comprehensive category encompassing the full range of digital content, platforms, and technological tools used in education. This approach ensures a holistic analysis by aligning with both international research paradigms and the specific policy framework governing the teachers in this study.

### UTAUT framework

The UTAUT (Unified Theory of Acceptance and Use of Technology) model provides a valuable perspective for studying teachers’ acceptance and use of new technologies in education and Psychology [27]. Proposed by Venkatesh et al. in 2003 [15], it integrates multiple theories on information technology adoption and use. Combining the strengths of eight models—Theory of Reasoned Action (TRA) [28], Theory of Planned Behaviour (TPB) [29], Technology Acceptance Model (TAM) [30], Motivational Model (MM) [31], Model of PC Utilisation (MPCU) [32], Combined TAM-TPB (C-TAM-TPB) [33], Innovation Diffusion Theory (IDT) [34], and Social Cognitive Theory (SCT) [35]—it offers a comprehensive framework for effectively explaining and predicting individuals’ acceptance and use of technologies, systems, and resources.

The original UTAUT model includes four main factors that directly influence behaviour: Performance Expectancy(PE), Effort Expectancy(EE), Social Influence(SI), and Facilitating Conditions(FC). Performance Expectations(PE) and Behavioral Intentions(BI) have been identified as the foremost predictive factors [27]. However, in the wide application of the UTAUT model, there is still controversy over the consensus on the validity of its core predictors, especially in terms of EE and FC. Firstly, the predictive ability of EE shows instability in various scenarios. Research shows that whether it is the adoption of online banking in the business field [36], the use of emergency information in the medical service field [37], or the integration of teacher technology in the education field, the direct impact of EE on user BI often weakens or even disappears [38]. Secondly, the theoretical approach of FC also faces challenges. Its direct driving effect on UB and BI is not static. For instance, in a study on the integration of digital collaboration technologies for teachers, FC significantly influenced BI, but no significant impact on UB [38], while in a study on the integration of technologies for pre-service teachers, its direct impact on UB has not been confirmed [39]. These contradictory findings suggest that specific situational factors may have altered the mechanism of action of the original variables in the UTAUT model. Therefore, this study holds that, in the context of government-supported longitudinal training, it is particularly important to test the applicability of the UTAUT model and possibly expand the moderating variables to explore how its factors function.

In recent years, researchers have adjusted these variables in studies to enhance the credibility of their findings. Wan Liyong and Zhao Chengling replaced “experience” and “voluntariness” with “educational background” when studying factors influencing the adoption and use of information technology by primary and secondary school teachers in Ethnic region areas [40]; Fang Xu et al. replaced “age” with “teaching experience” in their study on factors influencing teachers’ behaviour on STEM online education platforms [41]; Li Yi et al. introduced “regional differences,” “urban-rural differences,” and “inter-school differences” when analysing the factors and moderating effects of teachers’ use of information technology [42]; Guo Jiong and Fu Rui added “education level” and “teaching experience” as new moderating variables when exploring factors influencing rural teachers’ use of the “National Smart Education Platform for Primary and Secondary Schools” [43]. Adjusting the moderating variables of the UTAUT model in different contexts allows for a more accurate reflection of specific environments and populations, enhancing the depth and reliability of research. Notably, Zhao Duqing and Yu Liang, through a first-order meta-analytic structural equation model, analysed 56 studies and 61 independent correlation matrices to explore the expansion of the UTAUT and the substitution of its moderating variables. The study found that “region” significantly moderates the effect of “SI” on “BI”, while “education level” significantly moderates the relationship between “PE” and “BI [44]. Based on these previous studies, the necessity of considering context in the UTAUT model was emphasized. We initially determined to expand the basic framework by selecting “region”, “education level”, “gender” and “digital technology experience” as moderating variables. In the subsequent hypothesis development section, we will systematically expound why these specific factors are crucial for understanding the adoption intentions of K-12 teachers after longitudinal training.

### Theoretical framework and hypothesis development

**Fig 1:**
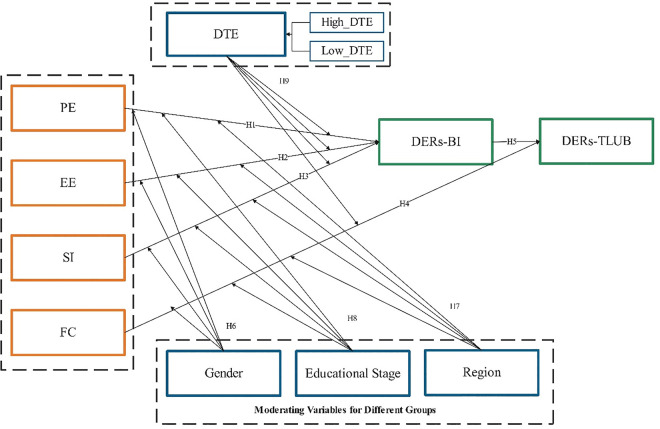
Extended moderating variables in the UTAUT model.

This study uses the UTAUT model to develop a framework for analyzing the factors influencing K-12 teachers’ adoption of DERs for teaching. The final modifications to the model in the study are shown in Fig 1. The core constructs and their application in this research are defined as follows:

### Direct effects of UTAUT constructs

PE is generally defined as the degree to which an individual believes that using a system will help them attain gains in job performance [15]. In this study, PE refers specifically to the extent to which K-12 teachers believe that using DERs will enhance their teaching performance. This includes the perception that DERs can lead to better student engagement, improved learning outcomes, and greater instructional efficiency. It is hypothesized that when teachers perceive a high utility in DERs, their intention to use them will increase. The following hypotheses are proposed:

**H1:** PE has a significant positive impact on the BI of K-12 teachers to use DERs for teaching.

EE is the degree of ease associated with the use of a system [45]. For this research, EE represents the level of effort teachers perceive is required to use DERs effectively in their classrooms. This encompasses the ease of finding, learning, and integrating these digital tools into their existing pedagogical practices.

**H2:** EE has a significant positive impact on the BI of K-12 teachers to use DERs for teaching.

SI refers to the degree to which an individual perceives that important others believe they should use a new system [15,45]. Within this study’s context, SI is the extent to which teachers feel influenced by the opinions and actions of their colleagues, school administrators, and professional peers regarding the adoption of DERs.

**H3:** SI has a significant positive impact on the BI of K-12 teachers to use DERs for teaching.

FC are the objective factors in the environment that an individual believes will support their use of a system. Here, FC refers to the tangible support provided by governments and schools, such as official policies, reliable technological infrastructure, technical assistance, and institutional incentives that encourage teachers to use DERs. The presence of strong FC is expected to remove barriers and enable the actual use of technology.

**H4:** FC has a significant positive impact on the UB of K-12 teachers using DERs.

BI refers to a person’s conscious willingness to engage in a specific activity [15]. It captures the degree of readiness to adopt a behavior and serves as a direct antecedent of actual UB. In this study, DERs-BI is the stated willingness of teachers to use DERs for their teaching. A strong BI is a primary predictor of actual technology use. Use Behavior, referred to as Teaching and Learning Use Behavior (DERs-TLUB), is the actual application of DERs by teachers in their classroom practices.

**H5:** The BI of K-12 teachers to use DERs can positively promote their UB.

### The moderating role of contextual factors

To exploring the heterogeneity of the K-12 teachers, this study extends the UTAUT model by incorporating gender, region, educational stage, and digital technology experience as moderating variables for a more nuanced analysis. A one-size-fits-all approach to technology integration is insufficient because the factors driving adoption are not universal but are instead shaped by the professional and social contexts of different teacher subgroups. The theoretical justification for our key moderators is as follows:

The choice of ‘region’ is crucial for addressing China’s significant “digital divide” [46], a challenge that resonates globally. Some research confirms that regional context moderates the effect of social influence on behavioral intention [42,44], and there are also differences in the application of information and communication technology infrastructure in primary and secondary schools in China [47]. Furthermore, studies have found that there are differences in belief and knowledge factors between urban and suburban areas for pre-service teachers during the process of technology integration, which also provides strong evidence for regional differences [48].

The “Education Stage” is included because the teaching objectives and curriculum requirements of primary, junior high and senior high schools vary greatly. This is a standard issued by the Ministry of Education of the country [49], which may affect teachers’ integration of DERs and digital technology. A previous study found that there are differences among primary school, junior high school and senior high school teachers in their perception and use of technology [50]. Furthermore, teachers at different educational stages often have differences in their TPACK capabilities [51,52], and these differences may lead to an impact on their integration in technology [53]. Overall, the research attention on the relationship between teachers’ expectations of technology adoption and behaviors at different educational stages is insufficient, especially in the context of this study..

Finally, this study refines the “experience” variable in the original UTAUT model into “digital technology experience”. In previous literature on the acceptance of educational technology, the “number of years of teaching experience” in educational work is often used as a proxy for the moderating effect of experience. Experience is an important moderating factor influencing the relationships among constructs such as performance expectations (PE), facilitating conditions (FC), relative advantages (RA), perceived usefulness (PU), and subjective norms (SN), supporting the theoretical basis. [54–56]. Based on this, subsequent research suggests that the quality and nature of the experience can be further emphasized by distinguishing between “novice” and “competen” users [57,58]. This method goes beyond time measurement to measure the functional digital readiness status of teachers. The methodological basis of this grouping is supported by those studies that have successfully used statistical boundaries to distinguish user groups based on user interaction frequency and skill levels [59]. This improvement enables a more precise study of how teachers’ existing digital capabilities affect their cognitive and support needs in the post-training environment. Based on this, the following hypotheses are proposed.

### Gender as a moderating hypothesis

**H6a:** Gender significantly moderates the relationship between PE and BI.**H6b:** Gender significantly moderates the relationship between EE and BI.**H6c:** Gender significantly moderates the relationship between SI and BI.**H6d:** Gender significantly moderates the relationship between FC intention and UB.

### Region as a moderating hypothesis

**H7a:** Region significantly moderates the relationship between PE and BI. **H7b:** Region significantly moderates the relationship between EE and BI. **H7c:** Region significantly moderates the relationship between SI and BI. **H7d:** Region significantly moderates the relationship between FC and UB.

### Educational stage as a moderating hypothesis

**H8a:** Educational stage significantly moderates the relationship between PE and BI. **H8b:** Educational stage significantly moderates the relationship between EE and BI. **H8c:** Educational stage significantly moderates the relationship between SI and BI. **H8d:** Educational stage significantly moderates the relationship between FC and UB.

### DTE as a moderating hypothesis

**H9a:** Experience significantly moderates the relationship between PE and BI. **H9b:** Experience significantly moderates the relationship between EE and BI. **H9c:** Experience significantly moderates the relationship between SI and BI. **H9d:** Experience significantly moderates the relationship between FC and UB.

## Methodology

### Instruments

Drawing on the measurement scales developed by Venkatesh et al. and from literature [60–64], this study adapts and refines survey items to align with the unique characteristics and practical realities of teachers’ use of DERs in China. The adaptation process involved two key stages. First, established items were translated into Chinese using standard back-translation procedures to ensure linguistic and conceptual equivalence. Second, these items were rephrased to specifically address the post-training context of K-12 teachers. For instance, questions about “the system” were specified to refer to “Digital Educational Resources.” A panel of five educational technology experts reviewed the adapted items to establish content and face validity, ensuring their relevance and clarity for the target population. The full list of measurement items is provided in Appendix.

The first section collects basic demographic information about teachers, including gender, teaching experience, region, educational level, and subject area. The second part consists of four questions, which are used to assess teachers’ experience level in digital technology (DERs-DET). The third part uses the UTAUT model to measure the influence of constructs such as SI, PE, EE, FC and BI on teachers’ actual der UB, including a scale of 17 items. The fourth part developed a 28-item scale based on the “Industry Standard for Digital Literacy of Chinese Teachers” to measure the digital literacy of K12 teachers, but this part was not used in this study. All items used in the present study were translated into Chinese using standard back-translation procedures with five-point Likert scale [65], with options ranging from 1 to 5, representing “Strongly Disagree,” “Disagree,” “Neutral,” “Agree,” and “Strongly Agree,” respectively.

### Procedures

To comprehensively explore the determinants of teachers’ behavioral intention to adopt DERs in classroom practice, this study was embedded within the framework of the 2024 Guangxi Online Training Programme on Digital Literacy Improvement for K-12 School Teachers. This initiative represents a large-scale, government-supported professional development program designed to elevate teachers’ capacity to integrate digital tools and resources into subject-specific pedagogy. This specific context was selected because it provided a standardized and uniform professional development intervention for a large-scale sample, establishing a robust baseline for subsequently examining differences across various teacher subgroups.

**Fig 2:**
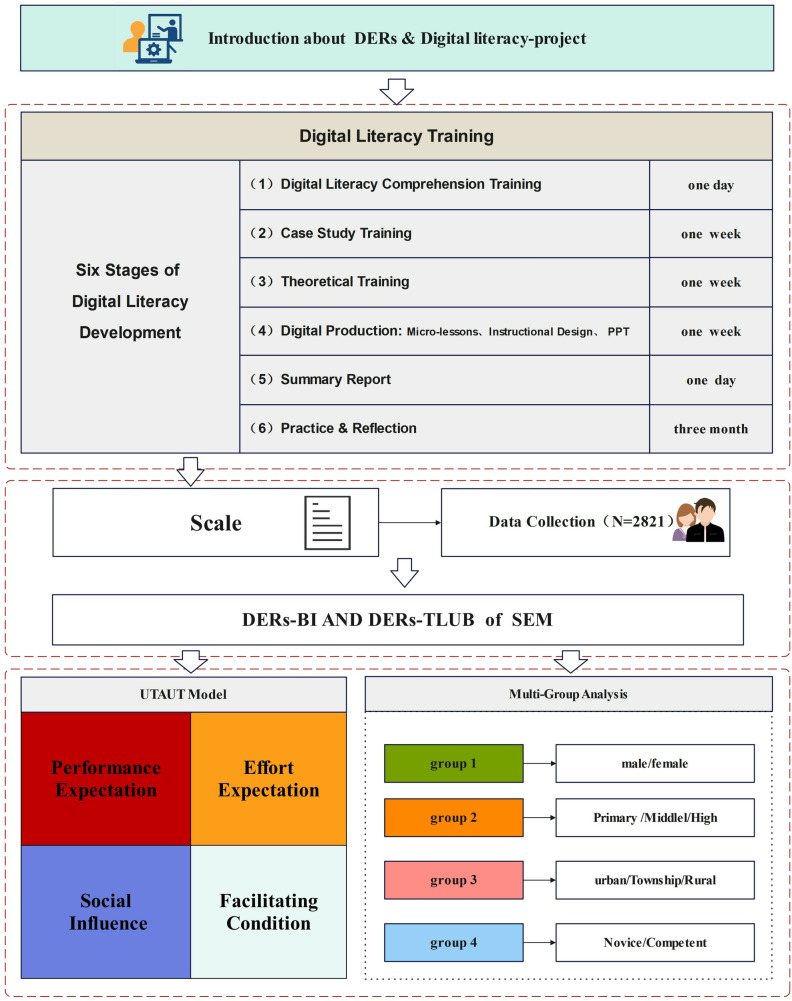
The procedure.

As illustrated in Fig 2, the research procedure followed a structured six-stage sequence, encompassing digital literacy training, classroom integration, and empirical evaluation, all grounded in the UTAUT framework.The training was systematically organized into six interconnected stages, each aligned with the goal of enhancing teachers’ engagement with DERs.

(1) Digital Literacy Concepts and Frameworks (1 day): This initial phase introduced participants to the conceptual foundations of digital literacy and the evolving landscape of digital education. Teachers explored core definitions, national policies, and international frameworks related to DERs. Special emphasis was placed on recognizing DERs as critical enablers of student-centered, competency-based learning in the digital era.
(2) Case-Based Application Training (1 week): Teachers engaged in guided analysis of best-practice cases featuring exemplary use of DERs across diverse disciplines and school contexts. Through group discussions and problem-based learning, they learned how to evaluate, select, and adapt existing DERs—including micro-courses, virtual labs, and online assessment tools—for authentic classroom scenarios.
(3) Theoretical Foundations of Digital Pedagogy (1 week): This stage deepened teachers’ understanding of pedagogical models underpinning DER integration, including TPACK (Technological Pedagogical Content Knowledge), SAMR, and constructivist learning theories. Teachers critically examined how DERs can enhance content delivery, formative assessment, and student engagement when aligned with specific teaching goals.
(4) Digital Resource Production and Instructional Design (1 week): Participants developed hands-on skills for creating digital educational resources using a range of authoring tools. Activities included designing multimedia micro-lessons, producing interactive slides, and curating open educational resources. Teachers also applied instructional design principles to ensure pedagogical alignment and accessibility of the DERs they produced.
(5) Digital Summary Reporting and Reflective Evaluation (1 day): Teachers synthesized their learning outcomes in the form of digital reflective reports, which included prototypes of DER-integrated lesson plans and micro-teaching demonstrations. Peer and facilitator feedback focused on the coherence between pedagogical intention, resource selection/design, and anticipated student learning outcomes.
(6) School-Based Implementation and Reflective Practice (3 months): Following the centralized training, teachers returned to their home schools to independently implement DER-based instruction. This extended practice phase served as a bridge between training and real-world integration. Teachers engaged in iterative teaching cycles using the DERs they had created or selected—ranging from digital reading materials and subject-specific software to AI-based assessment platforms. They also participated in school-based professional learning communities to share challenges and successes, further reinforcing the use of DERs in their subject teaching.

### Participants and data collection

The study’s participants were K-12 teachers drawn from a large-scale, government-endorsed digital literacy training program. The recruitment for the training program itself was intentionally designed using a stratified approach to ensure a representative cohort of teachers from across the Guangxi province [66,67]. Specifically, the program was made accessible to all active (non-retired) teachers from the province’s 14 cities, encompassing a full spectrum of geographic regions (urban, township, and rural), educational stages (primary, middle, and high school), and subject areas. To encourage broad participation, teachers were awarded continuing education credits upon completion of the training. This process ensured that the pool of trained educators, from which the survey sample was drawn, was demographically diverse and representative of the broader teacher population.

During the phase when teachers returned to schools to implement DERs-supported initiatives, all educators who participated in the digital literacy training were invited to voluntarily complete an anonymous online questionnaire. A total of 3,878 teacher responses were received. The survey was conducted on the popular Chinese platform www.wjx.cn and distributed via a link embedded within the SuperStar Learning Pass platform (a Chinese digital online learning platform). Teachers could continue using this platform for ongoing professional development after completing the training.The recruitment of participants for the survey commenced on 30/07/2024 and concluded on 30/08/2024.

To ensure data quality, rigorous exclusion criteria were applied. In James Soland’s research on identifying unengaged questionnaire responses, in fact, different questions should have different time thresholds. Responses within less than 2 seconds are mostly not serious enough and usually present as uniform answers [68]. As the study invited participants to fill out over 50 questions, which required varying amounts of effort, the final decision made by three educational technology researchers that responses to the scale could be completed within 90 seconds or less were excluded. Surveys were discarded if completed in under 90 seconds, exhibited uniform responses, or contained missing values. A final total of 2,821 valid responses were retained, yielding a response validity rate of 73%.

To confirm that the final samples obtained from the research are sufficient for the expected analysis, it is necessary to test its efficacy [69]. By using the proposed SEM prior power analysis sample size calculator [70]. Based on a significance level of α = 0.05, a statistical power of 0.90, a moderate effect size of 0.15, and a model structure with 6 latent variables and 17 observed variables, the minimum sample size required was calculated to be 2,136. Since the obtained samples easily exceeded this threshold, this study has sufficient statistical power to reliably detect significant effects. Table 1 provides detailed demographic data.

**Table 1:** Description of the study sample(N = 2821).

Category	Item	Number of People	Percentage(%)
Gender	male	729	25.8
	female	2092	74.2
Age	Under 30 years old	772	27.4
	31-40 years old	859	30.5
	41-50 years old	802	28.4
	Over 51 years old	388	13.8
Years of Teaching Experience	1-5 years	850	30.1
	6-10 years	461	16.3
	11-15 years	330	11.7
	16-20 years	238	8.4
	Over 20 years	942	33.4
Educational Qualification	Diploma or below	275	9.7
	Bachelor’s degree	2447	86.7
	Master’s degree or above	99	3.5
Professional Title	Junior (including unclassified)	274	9.7
	Intermediate	875	31
	Senior	1129	40
	Professor-level Senior	543	19.2
Teaching Subject	Chinese	638	22.6
	Mathematics	667	23.6
	Foreign Language	345	12.2
	Information Technology	203	7.2
	Others	968	34.3
School Region	urban	1312	46.5
	Township	1074	38.1
	Rural area	435	15.4
Educational Stage	Primary School	1110	39.3
	Middle School	913	32.4
	High School	798	28.3

### Ethical statement

Ethical approval for this study was granted by the Research Ethics Committee of the School of Computer and Information Engineering, Nanning Normal University (Protocol Code: NNUHR-2024-06), in compliance with the Declaration of Helsinki. Participation was entirely voluntary, and written informed consent was obtained from all individuals. Participants were informed that their involvement was anonymous and that all personal information would be handled with the strictest confidence, used solely for research purposes.

### Measures and statistical analysis

First, the internal consistency of the scale was established. Subsequently, the overall characteristics of the participants were described using descriptive statistics such as frequencies and percentages, while the study variables were calculated using means and standard deviations. IBM SPSS Statistics and AMOS 24 were used to assess the reliability and validity of the measurement model [71]. Based on Structural Equation Modeling (SEM), AMOS can test and validate the proposed model and examine relationships between key variables [72–74]. In addition, the extensive application of SEM in this field provides a solid basis and guidance for our methodological decisions [63,75–77]. Next, the structural equation model was used to evaluate model fit and test the research hypotheses related to factors influencing K-12 teachers’ use of DERs. Additionally, multi-group analysis was performed to explore how gender, educational level, region, and DERs-DET moderate the use of DERs.

## Results

### Digital technology experience of K-12 teachers

**Fig 3:**
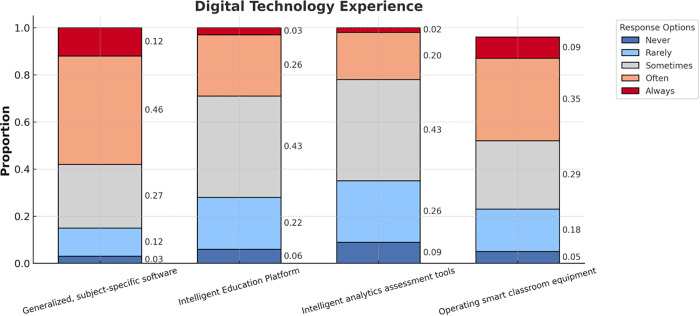
Digital technology experience.

The analysis of K-12 teachers’ experience with digital technology, presented in the stacked bar chart (Fig 3), reveals valuable insights into their engagement with four key digital tools: generalized, subject-specific software, intelligent education platforms, intelligent analytics assessment tools, and smart classroom equipment. The chart highlights that the majority of teachers report moderate use of these technologies, with the highest proportion indicating “Sometimes” or “Often” engagement. Specifically, generalized, subject-specific software shows the highest frequency of “Often” use (46%), followed by “Sometimes” (27%). Similar trends are observed for intelligent education platforms and intelligent analytics assessment tools, where moderate usage predominates. However, consistent use at higher frequencies, such as “Always,” remains relatively low across all categories, with only a small proportion of teachers reporting frequent use.

### Assessment of reliability and validity of the measurement model

We followed Hair et al. criteria to assess the reliability and validity of the measurement model [78]. The Cronbach’s alpha coefficients for all dimensions of the questionnaire exceed 0.8, with an overall coefficient of 0.958, indicating high reliability, which indicates acceptable internal consistency [79,80]. The Bartlett’s test of sphericity yielded a KMO value of 0.940 and a p-value < 0.001, indicating that the questionnaire is suitable for factor analysis.

Exploratory Factor Analysis (EFA) identified six principal components. After removing the low-factor-loading items SI3 and FC1, these six components aligned perfectly with the six latent variables designed in the questionnaire, indicating good structural validity. The survey instrument used in this study was adapted to the research context. Therefore, Confirmatory Factor Analysis (CFA) was conducted to further evaluate the convergent and discriminant validity of the measurement model. As shown in Table 2, all measurement items in the questionnaire have factor loadings greater than 0.7. The Composite Reliability (CR) values for all latent variables exceed 0.8, and the Average Variance Extracted (AVE) values are all above 0.5, indicating good convergent validity [81,82]. Furthermore, when the square root of the AVE for each latent variable is greater than its correlation with other latent variables, it demonstrates high discriminant validity [81]. In Table 3, the diagonal values represent the square root of the Average Variance Extracted (AVE) for each variable, while the values below the diagonal indicate the correlations between latent variables. The results show that the measurement model demonstrates good discriminant validity.

**Table 2:** Reliability and validity analysis of variables in the measurement model.

Construct	Indicator	M	SD	Cronbach’α	Factor Loadings	Composite Reliability	Convergence Validity
						CR	AVE
PE	PE1	4.29	0.728	0.94	0.921	0.86	0.95
	PE2	4.28	0.732		0.935		
	PE3	4.33	0.723		0.923		
EE	EE1	3.68	0.893	0.894	0.873	0.74	0.90
	EE2	3.83	0.877		0.884		
	EE3	3.62	0.905		0.831		
SI	SI1	3.87	0.864	0.883	0.889	0.83	0.91
	SI2	3.98	0.837		0.929		
FC	FC2	3.94	0.898	0.878	0.858	0.79	0.88
	FC3	3.93	0.87		0.92		
BI	BI1	4.2	0.757	0.949	0.943	0.88	0.96
	BI2	4.14	0.782		0.921		
	BI3	4.19	0.769		0.955		
UB	UB1	4.04	0.836	0.918	0.915	0.87	0.93
	UB2	4	0.859		0.948		

**Table 3:** Discriminant validity test of variables.

Construct	PE	EE	SI	FC	BI	UB
PE	0.975					
EE	.526**	0.947				
SI	.603**	.681**	0.954			
FC	.504**	.540**	.640**	0.938		
BI	.678**	.547**	.697**	.688**	0.980	
UB	.593**	.642**	.688**	.685**	.821**	0.964

Note: * indicates significance, where ** represents p ≤ 0.01 and *** represents p ≤ 0.001.

### Common method bias test

As the study relies entirely on questionnaires for data collection, common method bias (CMB) may be found in the studies [83], because both the independent variable and the dependent variable are captured by the same response method [84]. Therefore, Harman’s single-factor test was first used to examine common method bias across all items included in the hypothesis testing [85]. The first factor explains 36.905% of the total variance, which is below the 40% threshold. This indicates that there is no common method bias among the variables. Additionally, when two or more variables are highly correlated with each other, a correlation coefficient around 0.8 or 0.9 is considered excessive [86]. Scholars recommend using the variance inflation factor (VIF) to detect multicollinearity, as VIF quantifies the degree of correlation among predictor variables. Values exceeding 5 indicate severe multicollinearity, while values within the range of 3 ~ 5 suggest potential widespread threshold issues [87], with a commonly accepted threshold of 3.33 [88,89]. Consequently, VIF tests were conducted to ensure data accuracy. Results revealed VIF values ranging from 1.634 to 2.206 in this study, all falling below the specified threshold, further confirming the absence of multicollinearity issues.

### Model fit

Through the analysis of the sample data, it was found that the data does not conform to a multivariate normal distribution, and the sample size is large. Therefore, in SEM, the model fitting method in AMOS software uses Asymptotically Distribution-Free (ADF) [90,91], which is insensitive to the normality of the distribution of observed variables and is suitable for large sample sizes. In the model fit test, the suitability of the model is evaluated by analysing the fit indices of different structural variables. Higher fit indices indicate stronger explanatory power of the paths and greater significance of the model parameters. The results show that the CMIN/DF value of the model is relatively high, primarily due to the large sample size in this study, but it remains within an acceptable range [92,93]. Meanwhile, other commonly used fit indices (e.g., RMSEA, RMR, GFI, NFI, AGFI, EVCI, CFI) all meet the required standards, indicating good model fit [71,94]. Therefore, the model reasonably reflects the factors influencing the willingness of K-12 teachers to use DERs for teaching, providing a basis for related predictive work. Based on Wu Minglong’s model fit standards, the standardised parameter outputs for the hypothesised model in this study are shown in Table 4.

**Table 4:** CFA statistics of model fit.

	Statistical Test Value									
	Comprehensive Fit Index				Absolute Fit Index				Incremental Fit Index	
Indicators	CMIN/df	PGFI	PNFI	RMR	GFI	RMSEA	AGFI	EVCI	CFI	IFI
Critical Value	2 ~ 5	>0.50	>0.50	<0.05	>0.9	<0.10	>0.9	Minimize it	>0.9	>0.9
Model Value	3.363	0.599	0.651	0.026	0.947	0.029	0.916	0.122	0.926	0.927

### Hypothesis testing

**Fig 4:**
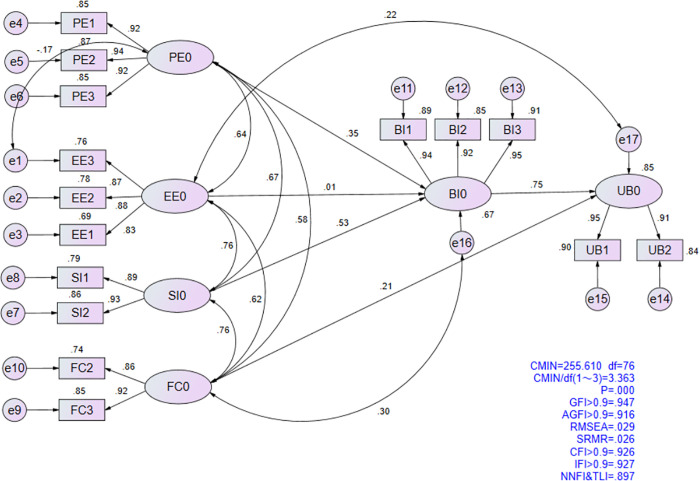
Standardized output results of the hypothetical model.

The structural equation model (SEM) was employed to test the direct effect hypotheses. The standardized output results from AMOS are presented in Fig 4, with specific validation details provided in Table 5.

**Table 5:** Path coefficient test results.

Hypothesis	Hypothesis Path	Standardized Path Coefficient	S.E.	C.R.	P	Result
H1	PE → BI	0.349	0.027	13.641	***	Supported
H2	EE → BI	0.011	0.023	0.451	0.652	Not
H3	SI → BI	0.531	0.029	16.962	***	Supported
H4	FC → UB	0.205	0.032	6.105	***	Supported
H5	BI → UB	0.751	0.033	23.884	***	Supported

Note: * indicates significance, where ** represents p ≤ 0.01 and *** represents p ≤ 0.001.

The results for the five proposed main effect hypotheses are as follows: PE (β = 0.349, p < 0.001) and SI (β = 0.531, p < 0.001) have a significant positive impact on BI (teachers’ willingness to apply DERs in teaching), with SI exhibiting the strongest effect. In contrast, EE (β = 0.011, p > 0.05) shows no significant impact on BI among teachers. Thus, hypotheses H1 and H3 are supported, while H2 is not supported. Additionally, FC (β = 0.205, p < 0.001) and BI (β = 0.751, p < 0.001) demonstrate a significant positive impact on UB (the application of DERs in teaching by K-12 teachers).

The explanatory power (R²) of the endogenous variable is a key criterion for evaluating the structural model [95]. The results support hypotheses H4 and H5, with the model accounting for 63.8% of the variance in UB (R² = 0.638), underscoring BI as the primary predictor in educational contexts.

### Multi-group analysis of moderating variable differences

To further explore how individual teacher differences impact the willingness and behaviour to apply digital education resources in teaching, the proposed moderating effect hypotheses were tested. The study grouped individual variables by different levels: teachers were divided into male and female groups by gender, and into urban, township, and rural groups based on the geographic location of their schools. Based on their experience with digital technology, participants were classified into “novice” and “competent” groups. Following Kelley’s theory of the 27% and 73%, the sample was divided into two groups to maximise differentiation [59].Teachers were grouped by educational level into primary, junior high, and senior high school groups. Amos multi-group analysis was used to explore the moderating effects of these individual variables, with pairwise comparisons conducted to examine differences in path coefficients between groups.

### Testing the moderating effect of gender

Under the premise of acceptable model fit, the critical ratio of path coefficient differences between groups is used to determine whether the path coefficients differ significantly. This tests whether nominal variables, such as gender, moderate the corresponding path relationships. The criterion is based on the “Critical Ratios for Differences Between Parameters” output in AMOS 24.0. Post-hoc tests assess whether the absolute value of the critical ratio for path coefficient differences between paired groups exceeds 1.96. If it does, the path coefficients show a significant difference [96]. From the direct effect test results, EE does not have a significant direct impact on BI (H2), and thus H6b, H7b, H8b, and H9b are not supported. As a result, their moderating effects are not tested further.

By constraining the path coefficients of the structural model to create a nested model, the difference in chi-square values between the nested model and the baseline model is tested for comparison. The results show that both the unconstrained model (χ2/df = 2.163, RMSEA = 0.020, CFI = 0.938, NFI = 0.893, IFI = 0.939) and the nested model (χ2/df = 2.119, RMSEA = 0.020, CFI = 0.937, NFI = 0.889, IFI = 0.938) exhibit good fit. The chi-square difference between the two models does not reach a significant level [Δχ2(9)=12.454, P = 0.189 > 0.05], indicating no significant difference in the structural paths of the model based on gender. This suggests that gender does not have a moderating effect on the structural paths of the model. The results of the gender moderation effect test are shown in Table 6.

**Table 6:** Moderating effect test results of gender.

Path Description	Critical Ratio	Hypothesis	Hypothesis Supported or Not
	Male Teachers/ Female Teachers		
PE → BI	−0.447	H6a	Not
EE → BI	−0.076	H6b	Not
SI → BI	0.433	H6c	Not
FC → UB	0.249	H6d	Not

### Testing the moderating effect of school location

Table 7 presents the critical ratios of path parameters among urban, township, and rural school groups, along with the test results for hypotheses H7a, H7b, H7c, and H7d. The absolute critical ratio of the path coefficient for PE → BI is 2.009 > 1.96, indicating a significant difference between urban and rural schools in the impact of PE on BI. Similarly, the absolute critical ratio of the path coefficient for FC → UB is 2.900 > 1.96, showing a significant difference between township and rural schools in the impact of FC on UB. Hypotheses H7a and H7d are supported, indicating that regional factors moderate the effects of PE on BI and FC on UB. Further comparison of path coefficients shows that, compared to urban school teachers, rural school teachers’ BI n is more strongly influenced by PE (β = 0.441, p < 0.001). Meanwhile, township school teachers are more strongly influenced by FC in UB (β = 0.425, p < 0.001).

**Table 7:** Moderating effect test results of school region groups.

Path Description	Critical Ratio			Hypothesis	Hypothesis Supported or Not
	urban/Township	urban/Rural	Township/Rural		
PE → BI	0.660	2.099*	1.532	H7a	Supported
EE → BI	−0.009	−0.860	−0.815	H7b	Not
SI → BI	0.244	0.581	0.385	H7c	Not
FC → UB	1.127	−1.958	−2.900**	H7d	Supported

### Testing the moderating effect of educational level

Table 8 presents the comparison of critical ratios for path parameters among primary, junior high, and senior high school teacher groups, along with the validation results for hypotheses H8a, H8b, H8c, and H8d. The study found that in the groups of primary and junior high school teachers, as well as junior and senior high school teachers, the absolute critical ratios for the path coefficients of PE → BI were 2.890 and 2.114, respectively, both exceeding 1.96. Additionally, the absolute critical ratio for the path coefficient of SI → BI was 2.794, also exceeding 1.96. This indicates that the effects of PE and SI on BI differ significantly between primary and junior high school teacher groups. The results for H8a and H8c further indicate that the moderating effects of PE and SI vary significantly across educational levels. A comparison of path coefficients reveals that junior high school teachers’ BI (β = 0.476, p < 0.001) is more strongly influenced by PE compared to primary school teachers (β = 0.372, p < 0.001) and senior high school teachers (β = 0.366, p < 0.001). In contrast, primary school teachers’ BI (β = 0.579, p < 0.001) is more significantly influenced by SI than that of junior high school teachers (β = 0.426, p < 0.001).

**Table 8:** Group moderation effect test results of educational stage.

Path Description	Critical Ratio			Hypothesis	Hypothesis Supported or Not
	Primary/Middlel	Primary/High	Middlel/High		
PE → BI	2.890**	0.774	−2.114**	H8a	Supported
EE → BI	0.944	1.245	0.414	H8b	Not
SI → BI	−2.794**	−1.734	0.756	H8c	Supported
FC → UB	0.449	0.961	0.690	H8d	Not

### Testing the moderating effect of experience

**Fig 5:**
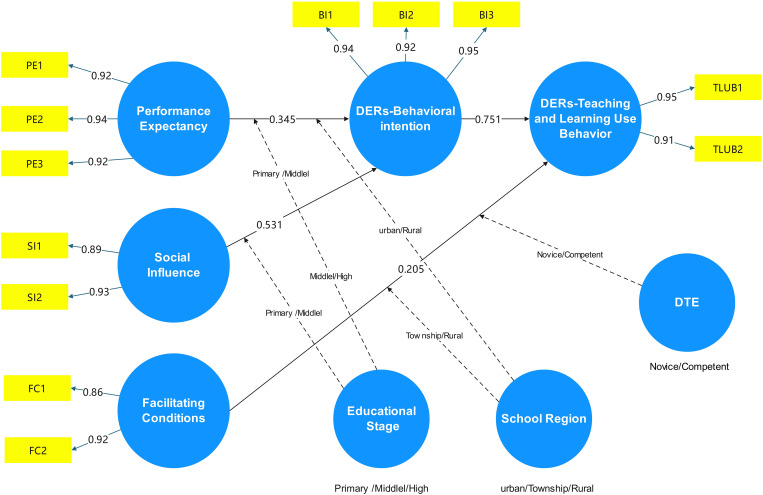
The final study.

Table 9 presents the group comparison between “novice” and “competent” teachers based on their level of digital technology experience. It includes the critical ratios of path parameters and the validation results for hypotheses H9a, H9b, H9c,and H9d. The analysis shows that the absolute critical ratio for the path coefficient of FC → UB is 2.007, exceeding 1.96. This indicates a significant difference in the impact of FC on UB between “novice” and “competent” teachers. The validation of H9d supports this conclusion, demonstrating that the level of digital technology experience moderates the effect of FC on UB. Further analysis of the path coefficients reveals that “competent” teachers (β = 0.526, p < 0.001) are more influenced by FC in UB than “novice” teachers (β = 0.473, p < 0.001).The final structural model is shown in Fig 5.

**Table 9:** Group moderation effect test results of experience.

Path Description	Critical Ratio	Hypothesis	Hypothesis Supported or Not
	Novice/Competent		
PE → BI	0.465	H9a	Not
EE → BI	−0.076	H9b	Not
SI → BI	−1.015	H9c	Not
FC → UB	−2.007*	H9d	Supported

## Discussion

This study investigated the factors shaping K–12 teachers’ intentions to DERs following their participation in a structured digital literacy program. Three key findings emerge. First, SI and PE were the primary determinants of teachers’ BI, whereas EE was not a significant predictor. Second, while FC had a direct effect on usage, behavioral intention proved to be the most powerful predictor of actual UB; together, these factors explained a substantial portion of the variance (R² = 0.638). Third, and critically, these relationships were not universal. The influence of the core UTAUT predictors was significantly moderated by the teachers’ regional context, educational stage, and prior digital technology experience, though not by gender. These findings collectively challenge the one-size-fits-all approach to technology integration, revealing that technology use involves diverse paradoxes influenced by complex interactions among psychological, contextual, and technological factors [97,98]. They underscore the importance of supportive structures and differentiated strategies, offering a more nuanced explanation for the mixed outcomes of large-scale teacher training programs.

### The analysis of supported hypotheses

These UTAUT path relationships refine expectations for school settings. The SI → BI path was strongest, consistent with evidence that teachers respond to norms, leadership expectations, and professional communities [27]. After vertical digital literacy training, the SI → BI path remains the strongest., the SI → BI path remained the strongest. This dominance, which contrasts with standard UTAUT expectations of a more balanced set of predictors, suggests that teachers’ adoption intentions were not formed in isolation but were profoundly shaped by the study’s specific context. The large-scale, government-endorsed nature of the training program likely created significant systemic pressure and established powerful professional norms, amplifying the role of social influence.This explanation is consistent with an increasing number of studies. The research results of Hamamra, Khlaif and their colleagues emphasize how institutional culture and structural factors influence educators’ experiences of technology integration [99,100]. Similarly, in Khlaif’s research, it is believed that insufficient school support can affect their level of technological stress, thereby influencing the integration of new technologies [98]. This suggests that teachers’ intentions to adopt DERs were shaped mainly by the expectations and behaviors of colleagues and school leaders, consistent with prior findings on the role of social influence in technology adoption [7,55]. The PE → BI path was also significant: teachers intend to use DERs when they expect gains in instruction and student learning, as UTAUT predicts [15].The relative strength of SI and PE, juxtaposed with the non-significance of EE, points to a decision-making context where professional alignment and practical outcomes are valued more than the effort required for adoption.

Our multi-group analysis revealed important nuances. The finding that the PE → BI relationship was stronger for rural teachers than for their urban counterparts,Compared with urban students, receiving higher education is one of the few ways for rural students to succeed. Therefore, rural education places more emphasis on the college admission rate than urban education. Under the premise that information technology can increase the college admission rate, rural teachers will be more willing to use information technology for teaching [42]. This is one reasonable explanation in the Chinese context, suggests that rural educators may be particularly motivated by the potential of DERs to overcome resource limitations and improve instructional quality. Furthermore, the moderating effects of educational stage and experience highlight context-specific needs. For example, the influence of SI → BI was stronger for primary school teachers, who may work in more collaborative, team-oriented environments, in the educational environment of China, primary and secondary school teachers, due to their stronger group and collaborative nature, are more susceptible to the influence of community norms and peer demonstrations. However, junior high school teachers have high independence and strong technical effectiveness, and their community influence is relatively weak. This complex relationship can be further explored in the future. The impact of FC → UB was stronger for teachers with more DTE, this indicates that when teachers already possess basic digital skills, their technical stress and anxiety will be at a relatively low level [98], reliable infrastructure and technical assistance and other institutional supports are the most effective. These differences help explain the mixed results often seen in national technology initiatives and underscore the challenge of ensuring equitable DERs integration [14].

### The analysis of unsupported hypotheses

One of the study’s notable findings was the lack of a significant relationship between EE and BI (H2), which contrasts with the original UTAUT model but is not unprecedented in previous research [36–38,101]. In Gruzd [102] and Dwivedi’s [103] understanding of users’ adoption of technical tools, EE is regarded as a key obstacle. An individual’s attitude may be influenced by the degree to which the technology is easy to use (i.e., less complex) and whether the technology has proven useful (i.e., higher performance), and the adoption of trained DERs, EE is no longer a difficulty for teachers, which can reasonably explain this inconsistency. After completing intensive training, teachers’ concerns about the difficulty of using DERs may become less important than their own self-efficacy and the expectations of their peers [104]. These outcome suggests that the mandatory, intensive training program preceding our data collection is central to the explanation. Once teachers are guided past initial usability challenges and develop a baseline digital competency, their decision-making calculus appears to shift. The focus moves from the ease of use to the technology’s instrumental value, the prevailing social norms and facilitating conditions. We posit that the standardized training homogenized teachers’ perceptions of difficulty, thereby reducing the variance in EE across the sample and diminishing its predictive power in the model.

Several moderation hypotheses were also unsupported. Gender did not moderate any path, This is consistent with the important research results on technology acceptance in some past educational fields [105,106], suggesting that once access and training are provided at scale, both male and female teachers have received adequate training gender-based differences in adoption drivers diminish. Educational stage did not moderate FC → UB, and region did not moderate SI → BI, these null results are informative: they imply that building dependable infrastructure and platform support is universally beneficial across stages [103], and the social expectations teachers perceive around using DERs look similar across regions in this sample. Overall, the unsupported hypotheses narrow the areas where tailored approaches are necessary—PE in rural areas, SI in primary schools, and FC for experienced users.

### Theoretical implications

From a theoretical standpoint, this study provides a critical extension to the technology acceptance literature, particularly the UTAUT framework within educational contexts. Its primary contribution lies in challenging the common methodological assumption that treats teachers as a single, uniform group [7,107]. This is a significant advancement, as many previous studies have applied only a subset of the UTAUT model and have often omitted the vital role of moderating variables [45]. In the current landscape of global education digitalization, where DERs are increasingly central to national strategies, such a comprehensive approach is paramount. Without accounting for moderated effects, theoretical models can fail to explain why large-scale technology initiatives yield inconsistent results.

By analyzing the full UTAUT model across distinct teacher subgroups, our findings demonstrate that the predictive power of its core constructs is not universal. Instead, the determinants of DERs adoption are highly contingent on contextual factors such as regional setting, educational stage, and prior experience, even when all participants receive identical training. This research, therefore, underscores the necessity of integrating demographic and contextual moderators to enhance the explanatory power of technology acceptance models. It effectively moves the theoretical discourse from simply asking if teachers will adopt DERs to a more sophisticated and actionable inquiry into which teachers are likely to adopt them, under what specific conditions, and why.

### Practical implications

The results offer clear, actionable guidance for policymakers and school leaders aiming to design more effective and equitable technology integration strategies. The central takeaway is the need to move away from a single, standardized support model toward a more differentiated approach that aligns with the specific motivators of different teacher groups.

**For primary schools**, where Social Influence is a dominant driver, school leaders should foster adoption by cultivating peer-led learning communities, encouraging mentorship, and publicly celebrating innovative uses of **DERs** by respected colleagues [108].

In rural areas, where PE is paramount, professional development must focus on demonstrating the concrete and measurable benefits of DERs for improving student learning outcomes and overcoming resource limitations.

**Support should be customized based on digital competence.** For digitally experienced teachers, who are more influenced by FC, the priority should be ensuring access to high-quality digital content and providing responsive, reliable technical support. Conversely, for novice users, providing robust infrastructure is insufficient; they require structured, scaffolded coaching and curated resources to build confidence and bridge the gap between their intention to use **DERs** and their actual classroom practice.

Ultimately, policy investments must extend beyond platforms and devices. To ensure that funding for technology yields meaningful educational value, resources should be directed toward cultivating the sustained, collaborative professional structures necessary to maintain social momentum and provide clear evidence of instructional gains.

## Conclusion

Following structured digital literacy training, K-12 teachers’ decisions to use DERs are most strongly shaped by SI and PE. This intention, in turn, is the primary driver of their actual classroom practice. Our findings reveal that these effects are not uniform; they are significantly moderated by regional context, educational stage, and digital technology experience, but not by gender. The irrelevance of EE suggests that once teachers are trained, concerns about ease-of-use become secondary to the technology’s perceived benefits and prevailing social norms. The key contribution of this study is a nuanced understanding of these teacher differences, which clarifies why universal training initiatives yield uneven results and identifies where targeted support is most effective: fostering social proof in primary schools, demonstrating instructional gains in rural contexts, and ensuring dependable technical support for experienced educators..

### Limitations and future research

This study has limitations that offer avenues for future research. The findings are based on self-reported data from a single province, which may limit their generalizability. Future work should therefore extend this study to other regions and incorporate objective measures, such as classroom observations and student learning outcomes, to validate these findings. Additionally, experimental studies are needed to establish the causal effects of the tailored interventions suggested here. Despite these limitations, the central message for policymakers and practitioners is clear: to successfully turn training into practice at scale, it is essential to align social norms, demonstrate clear instructional benefits, and provide reliable support—all while tailoring these strategies to the diverse needs of teachers.

## Supporting information

S1 Data Raw data of this study. (XLSX)

S1 Appendix Questionnaire used for data collection, detailing all survey items. (DOCX)
