# Supplementary material for: From training to practice: A multi-group analysis of factors influencing K-12 teachers’integration of Digital Educational Resources (DERs)
Source: PLoS One. 2025 Dec 16;20(12):e0338543. doi: 10.1371/journal.pone.0338543 (PMC12707651; doi:10.1371/journal.pone.0338543)
Supplement: S1 Appendix — (DOCX) [file pone.0338543.s002.docx]

**Appendix:**

Questionnaire:

| ****Part**** | ****Item**** | ****Question**** | ****Answer Options**** | ****Cite**** |
| --- | --- | --- | --- | --- |
| **Part I. Teacher Demographic Information** | | | |  |
|  | 1 | Your gender | Male / Female | By Authors |
|  | 2 | Your age group | Under 30 / 31–40 / 41–50 / Above 50 |  |
|  | 3 | Your years of teaching experience | 1–5 years / 6–10 years / 11–15 years / 16–20 years / Over 20 years |  |
|  | 4 | Your educational background | Junior college or below / Bachelor’s degree / Master’s degree or above |  |
|  | 5 | Your professional title | Primary (unrated) / Intermediate / Senior / Professor-level senior |  |
|  | 6 | The subject(s) you teach | Chinese / Mathematics / Foreign Language / Information Technology / Others |  |
|  | 7 | The geographical location of your school | Urban / Town / Rural |  |
|  | 8 | The educational level you teach | Primary / Junior high / Senior high |  |
| **Part II. Teachers’**digital **Experience** | | | |  |
|  | DET1 | I have experience using general software and subject-specific software (such as Bagui Jiaoxuetong, Guijiaotong, or Xiwoboard) for teaching. | Not at all/Very few/Sometimes/Often/  Very frequently | By Authors |
|  | DET2 | I have experience using intelligent education platforms (such as the Autonomous Region Smart Education Platform or the National Smart Education Platform) for professional learning and development. | Same as above |  |
|  | DET3 | I have experience using intelligent analysis and evaluation tools for teaching and assessment. | Same as above |  |
|  | DET4 | I have experience operating smart classroom equipment for teaching. | Same as above |  |
| **Part III. Teachers’ Intention to Use Digital Educational Resources** | | | |  |
|  | PE1 | I find that digital educational resources help enrich my classroom teaching. | Strongly disagree – Disagree – Neutral – Agree – Strongly agree | [60][61]  [62][63]  [64] |
|  | PE2 | Using digital educational resources helps me organize, implement, and evaluate teaching more efficiently. | Same as above |  |
|  | PE3 | Using digital educational resources improves my teaching efficiency. | Same as above |  |
|  | EE1 | It is easy for me to learn how to use digital educational resources in teaching. | Same as above |  |
|  | EE2 | I think digital educational resources are easy to understand and apply in practice. | Same as above |  |
|  | EE3 | I find it simple to apply digital educational resources in teaching. | Same as above |  |
|  | SI1 | My colleagues around me are trying to apply digital educational resources to support teaching. | Same as above |  |
|  | SI2 | My colleagues or teachers encourage me to apply digital educational resources in teaching. | Same as above |  |
|  | SI3 | If my colleagues and teachers are using digital educational resources, I am likely to try them as well. | Same as above |  |
|  | FC1 | I can get technical support whenever I encounter problems in using digital educational resources for teaching. | Same as above |  |
|  | FC2 | My school provides sufficient hardware (such as computers and projectors) and software (such as teaching applications and platforms) to support the use of digital educational resources in teaching. | Same as above |  |
|  | FC3 | Educational authorities and my school support and encourage me to use digital educational resources in teaching and provide necessary conditions (such as training). | Same as above |  |
|  | BI1 | I plan to continue applying digital educational resources in my teaching. | Same as above |  |
|  | BI2 | I would suggest that my colleagues use digital educational resources in their teaching. | Same as above |  |
|  | BI3 | I will keep using digital educational resources to support my teaching. | Same as above |  |
|  | UB1 | I often apply digital educational resources in teaching or design teaching activities based on them. | Same as above |  |
|  | UB2 | I frequently use digital educational resources to support lesson preparation, reflection, and professional collaboration. | Same as above |  |
| **Part IV. Teachers’ Digital Literacy** | | | |  |
| **(I) Digital Awareness** | DA1 | I can recognize the importance of digital technologies (such as artificial intelligence, big data, and virtual reality) in the development of digital education. | Strongly disagree – Disagree – Neutral – Agree – Strongly agree | By Authors  (industry Standard Document for Digital Literacy of Chinese Teachers) |
|  | DA2 | I believe the development of digital technologies brings new opportunities and challenges to education and teaching. | Same as above |  |
|  | DA3 | I am willing to actively learn and use digital technology resources and apply them in education and teaching. | Same as above |  |
|  | DA4 | I am willing to integrate digital technologies with education and teaching to achieve innovation. | Same as above |  |
|  | DA5 | When encountering difficulties in digital education practice, I can actively seek solutions. | Same as above |  |
| **(II) Digital Technology Knowledge and Skills** | DTKS1 | I can understand the basic characteristics of common digital technologies (e.g., multimedia, the Internet, big data, virtual reality, artificial intelligence). | Same as above |  |
|  | DKTS2 | I can select and use digital technology resources appropriately according to different teaching goals and actual teaching situations. | Same as above |  |
|  | DTKS3 | I can skillfully use digital devices, software, and platforms commonly used in education and teaching. | Same as above |  |
| **(III) Digital Application** | DAP1 | I can use digital assessment tools to analyze students’ learning conditions (e.g., using automated grading systems, question banks, or evaluation systems to analyze students’ readiness, ability, and learning styles). | Same as above |  |
|  | DAP2 | I can obtain, manage, and create digital educational resources, and select or produce them according to teaching needs (e.g., collecting online materials to make multimedia lessons or micro-courses). | Same as above |  |
|  | DAP3 | I can design teaching activities that integrate digital technologies (e.g., using interactive whiteboards or tablets appropriately during lessons). | Same as above |  |
|  | DAP4 | I can use digital technology resources to create blended learning environments that combine online and physical spaces (e.g., using VR, AR, or MR technologies to overcome space-time limits). | Same as above |  |
|  | DAP5 | I can use digital technology resources to conduct teaching activities, optimize teaching processes, and enhance students’ participation and learning experience. | Same as above |  |
|  | DAP6 | I can use digital technology resources to observe students’ learning differences and provide targeted guidance. | Same as above |  |
|  | DAP7 | I can use data collection tools (e.g., online survey platforms or recording classrooms) to gather and analyze students’ academic data. | Same as above |  |
|  | DAP8 | I can use digital technology resources to conduct moral and mental health education and address related student issues in time. | Same as above |  |
|  | DAP9 | I can guide students to engage in digital learning and enhance their sense of digital social responsibility. | Same as above |  |
|  | DAP10 | I can use different digital communication tools to collaborate and communicate effectively with parents. | Same as above |  |
| **(IV) Digital Social Responsibility** | DSR1 | In the digital society, I can comply with Internet laws, regulations, and communication norms, consciously maintain proper online behavior, and help create a positive online environment. | Same as above |  |
|  | DSR2 | I can reasonably use digital products and services, follow principles of legitimacy, informed consent, clear purpose, and safety, respect intellectual property, and care for students’ mental and physical health. | Same as above |  |
|  | DSR2 | In the digital society, I consciously protect my personal data privacy during information sharing. | Same as above |  |
|  | DSR3 | In my work, I pay attention to data security when collecting, storing, using, and sharing information about students, parents, and others. | Same as above |  |
|  | DSR4 | I can effectively identify, prevent, and respond to online rumors, telecom fraud, and information theft. | Same as above |  |
| **(V) Professional Development** | PD1 | I can use digital technology resources to engage in continuous professional learning and development according to my own growth needs. | Same as above |  |
|  | PD2 | I can use digital technology resources (e.g., online lessons, open courses, and exemplary teaching videos) to reflect on and improve my teaching. | Same as above |  |
|  | PD3 | I can actively participate in online teaching and training activities, exchanging ideas and sharing teaching content and resources with others. | Same as above |  |
|  | PD4 | I can use digital technology resources to conduct research on issues related to digital teaching. | Same as above |  |
|  | PD5 | I can use digital technology resources to continuously explore innovations in teaching models and student learning approaches. | Same as above |  |
